# Clark’s Nutcracker Breeding Season Space Use and Foraging Behavior

**DOI:** 10.1371/journal.pone.0149116

**Published:** 2016-02-16

**Authors:** Taza D. Schaming

**Affiliations:** Department of Natural Resources, Cornell Lab of Ornithology, Cornell University, Ithaca, New York, United States of America; Estación Biológica de Doñana, CSIC, SPAIN

## Abstract

Considering the entire life history of a species is fundamental to developing effective conservation strategies. Decreasing populations of five-needle white pines may be leading to the decline of Clark’s nutcrackers (*Nucifraga columbiana*). These birds are important seed dispersers for at least ten conifer species in the western U.S., including whitebark pine (*Pinus albicaulis*), an obligate mutualist of Clark’s nutcrackers. For effective conservation of both Clark’s nutcrackers and whitebark pine, it is essential to ensure stability of Clark’s nutcracker populations. My objectives were to examine Clark’s nutcracker breeding season home range size, territoriality, habitat selection, and foraging behavior in the southern Greater Yellowstone Ecosystem, a region where whitebark pine is declining. I radio-tracked Clark’s nutcrackers in 2011, a population-wide nonbreeding year following a low whitebark pine cone crop, and 2012, a breeding year following a high cone crop. Results suggest Douglas-fir (*Pseudotsuga menziesii*) communities are important habitat for Clark’s nutcrackers because they selected it for home ranges. In contrast, they did not select whitebark pine habitat. However, Clark’s nutcrackers did adjust their use of whitebark pine habitat between years, suggesting that, in some springs, whitebark pine habitat may be used more than previously expected. Newly extracted Douglas-fir seeds were an important food source both years. On the other hand, cached seeds made up a relatively lower proportion of the diet in 2011, suggesting cached seeds are not a reliable spring food source. Land managers focus on restoring whitebark pine habitat with the assumption that Clark’s nutcrackers will be available to continue seed dispersal. In the Greater Yellowstone Ecosystem, Clark’s nutcracker populations may be more likely to be retained year-round when whitebark pine restoration efforts are located adjacent to Douglas-fir habitat. By extrapolation, whitebark pine restoration efforts in other regions may consider prioritizing restoration of whitebark pine stands near alternative seed sources.

## Introduction

For effective conservation, it is important to consider the entire life history of a species [1]. Understanding home range size, territoriality, habitat selection, and foraging behavior is fundamental to predicting a species vulnerability to decline [1,2]. It is also important to the development of management and conservation strategies [1]. In particular, the habitat selected during all important life stages should be considered when designing management plans. For example, neotropical migrants require both specific northern breeding and southern wintering habitats, and spotted salamanders (*Ambystoma maculatum*) breed in vernal pools, then use surrounding woodlands the remainder of the year [3,4]. Sound management strategies may depend on protection of multiple habitat types.

Decreasing populations of five-needle white pines may be leading to the decline of Clark’s nutcrackers (*Nucifraga columbiana*) in large parts of their range [5–8]. Previous research has revealed that in whitebark pine (*Pinus albicaulis*) habitat, the frequency of Clark’s nutcracker occurrence decreased with lower whitebark pine cone production [7,8]. Fewer five-needle white pines leads to fewer cones, which leads to fewer Clark’s nutcrackers. In areas where its primary seed sources are declining, Clark’s nutcrackers may increasingly need alternate seed sources and habitats to support populations.

Large-seeded pines are important foraging habitat for Clark’s nutcrackers, because each individual stores tens of thousands of conifer seeds every autumn [9,10]. The birds use the cached seeds for food for both overwinter survival and feeding nestlings, but are estimated to cache two to five times their energetic requirements [9–12]. The importance of specific large-seeded pines to Clark’s nutcrackers’ diet varies geographically, and all the pines are subject to years of low cone production [6,13,14]. When preferred pines produce few cones, alternative seed sources are essential. Clark’s nutcrackers may forage on less preferred local conifer species, or, in years with widespread cone crop failure, birds will move out of the ecosystem [15,16].

In many areas, Clark’s nutcrackers use whitebark pine seeds as an important food source [9,17]. Whitebark pine is a keystone species and an obligate mutualist of Clark’s nutcrackers [13,14]. It depends on Clark’s nutcrackers for dispersal of its wingless seeds [13,14]. This Clark’s nutcracker-whitebark pine mutualism is critical to ecosystem function [13,14]. Whitebark pines play an important role in providing important ecosystem services, including providing food and habitat for wildlife, preventing erosion and protecting watersheds [5,18–20]. Currently, whitebark pine forest communities are rapidly disappearing range-wide due to decades of fire suppression, widespread infection by the non-native fungal pathogen *Cronartium ribicola*, which causes white pine blister rust, and outbreaks of mountain pine beetles (*Dendroctonus ponderosae*) [13]. Consequently, extensive efforts are in place to restore whitebark pine, with the assumption that Clark’s nutcrackers will continue to be available to disperse the whitebark pine seeds [21]. It is vital to protect Clark’s nutcracker populations because they are important seed dispersers for not just whitebark pine, but for at least ten conifer species in the western U.S. [22]. The continued dispersal of pine seeds by Clark’s nutcrackers increases the regeneration capacity of the declining five-needle pines.

Previous research in whitebark pine ecosystems has documented the importance of multiple conifers, including ponderosa pine (*Pinus ponderosa*), Jeffrey pine (*Pinus jeffreyi*), and Douglas-fir (*Pseudotsuga menziesii*), to Clark’s nutcrackers during the autumn harvest season [17,22,23]. When whitebark pine cone crops are depleted, the birds begin harvesting other locally available seeds [17]. Clark’s nutcracker breeding season activities are also intimately linked to the autumn harvest. During the breeding season, seeds cached the previous autumn are consumed by adults and nestlings [11,24,25]. Also, in the Greater Yellowstone Ecosystem, where whitebark pine is the predominate large-seeded conifer, previous research suggests that Clark’s nutcracker populations do not breed in years following low whitebark pine cone crops [26]. Understanding Clark’s nutcracker breeding season space use and foraging behavior in whitebark pine ecosystems—particularly the variation between years following low vs. high whitebark pine cone crops—is essential to our understanding of how Clark’s nutcrackers can persist in these declining ecosystems. Despite its importance, Clark’s nutcracker breeding season space use is poorly studied [6,27].

Clark’s nutcracker breeding habitat varies geographically [6]. Clark’s nutcrackers breed in multiple forest communities including piñon-juniper woodland (*Pinus edulis* and *Pinus monophylla*, and *Juniperus* spp.), ponderosa pine, Douglas-fir, Jeffrey pine, and mixed coniferous subalpine communities which include whitebark or limber pine (*Pinus flexilis*) [See 6]. Observational studies suggest that during the breeding season, whitebark pine communities are used infrequently. Nonetheless, all of the breeding habitats used include conifer seed sources. In the only previous systematic study of space use of radio-tracked Clark’s nutcrackers, breeding season space use and foraging behavior were not separately evaluated [22,27].

My objectives were to evaluate Clark’s nutcracker breeding season home range size, territoriality, habitat selection, and foraging behavior in the southern Greater Yellowstone Ecosystem, a region with large-scale whitebark pine decline [28]. I assessed territoriality because evidence of territoriality would influence both home range size and habitat selection. I examined individual behavior over two years, a nonbreeding and a breeding year [26]. The nonbreeding year followed an autumn with a lower whitebark pine cone crop and had a higher spring snowpack compared to the breeding year [26]. By focusing on two years with diverse demographic and environmental conditions, I evaluated a wider range of behavioral responses. By working in a region with extensive mortality of whitebark pine, the results will aid in understanding the range of responses that Clark’s nutcracker populations exhibit as the habitats and the resources they provide are lost. This information will contribute to the creation of more effective management strategies.

## Materials and Methods

### Ethics Statement

I captured and handled all birds according to Animal Care Protocol guidelines approved by Cornell University. This research was approved by the Cornell University Institutional Animal Care and Use Committee (protocol # 2008–0176). I banded Clark’s nutcrackers under U.S. Fish and Wildlife Permit # 23533, and Wyoming Game and Fish Chapter 33 Permit # 695. I conducted all field work under U.S. Forest Service Special-Use Authorization # JAC747002 (2009–2013) and Grand Teton National Park Scientific Research and Collecting Permit #’s GRTE-2011-SCI-0052 and GRTE-2012-SCI-0069.

### Field Methodology

#### Study area

Between 2009 and 2015, I studied Clark’s nutcrackers in the southern Greater Yellowstone Ecosystem, primarily in Bridger Teton and Shoshone National Forests, and Grand Teton National Park (25,050 km^2^). This portion of the study is based on the years 2011–2012, the only years in which I intensively radio-tracked and conducted regular behavioral observations of radio-tagged Clark’s nutcrackers. It was predominantly conducted in the area bounded by 43°56’10” N north, 43°34’34” N south, 110°38’20” W west, and 110°04’59” W east (~1,220 km^2^). The forested habitat primarily consists of six conifer species: whitebark pine, limber pine, Douglas-fir, lodgepole pine (*Pinus contorta*), Engelmann spruce (*Picea englemannii*), and subalpine fir (*Abies lasiocarpa*). The conifer habitat is intermixed with aspen (*Populus tremuloides)*, sagebrush (*Artemesia tridentata*), grassy open areas, high mountain meadows and rocky outcroppings.

#### Seasonal boundaries used in this study

I based seasonal boundaries on breeding years 2010 and 2012 because I did not observe breeding Clark’s nutcrackers in my study area in 2011. The prebreeding season ranged from January 15, the first date I trapped Clark’s nutcrackers, through March 4. The breeding season is considered March 5, the earliest date I observed a Clark’s nutcracker building a nest during the study, through June 15, the last date I observed a nestling on a nest (S1 Fig). The seed preharvest season is the time period during which Clark’s nutcrackers were eating immature whitebark pine seeds, but not yet caching mature seeds. It began June 16 and ended the day prior to my first observation of a Clark’s nutcracker with a full sublingual pouch each year, August 8, 2011 and July 29, 2012.

#### Capture and marking

**Fig 1:**
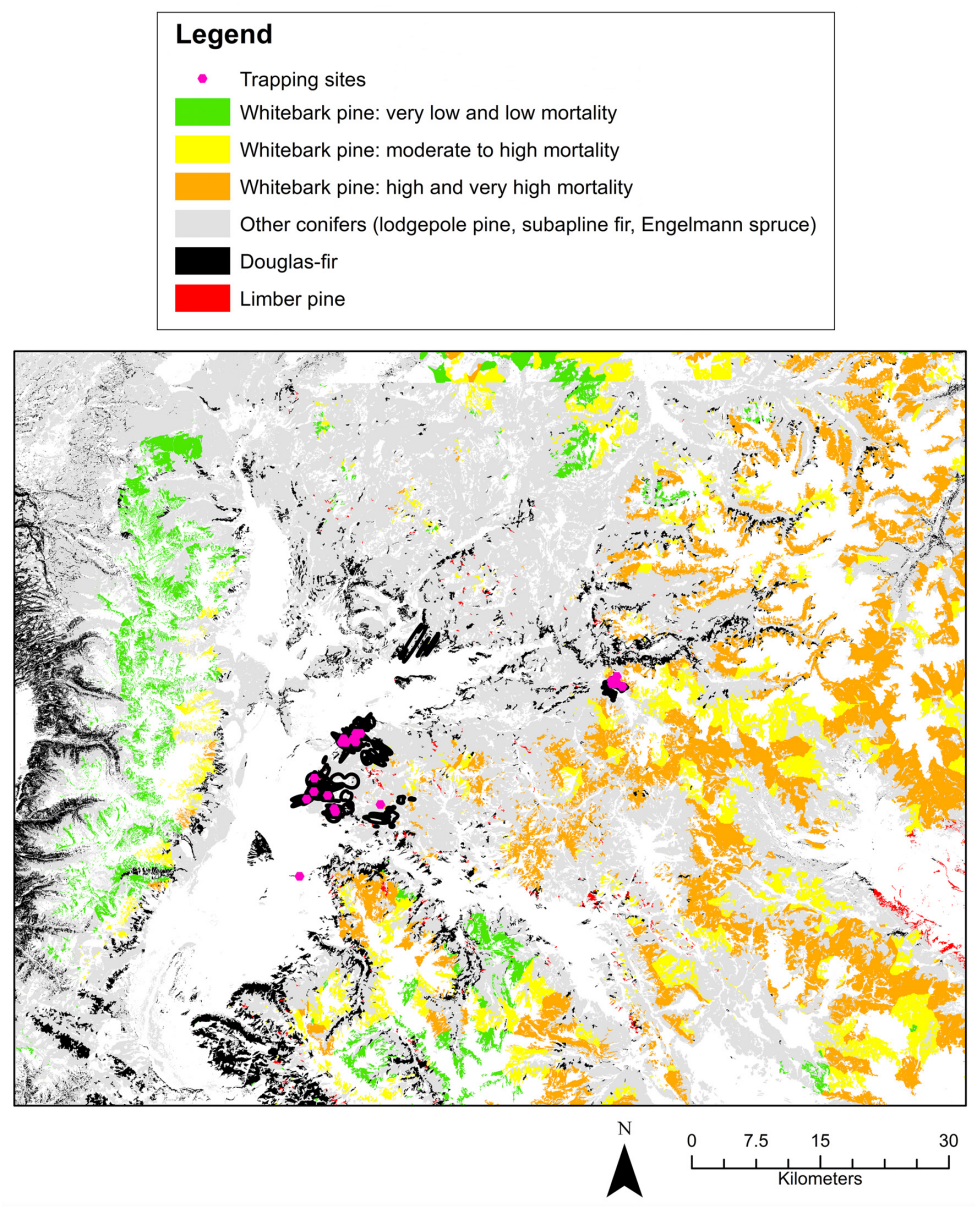
Trapping locations, individual home ranges, and available habitat. Only trapping sites where individuals were successfully captured are shown. Specific whitebark pine categories were merged for better visualization. Burned habitat is not shown because it is not possible to see at the scale of the map.

Each year, I located trapping sites for radio-tagging Clark’s nutcrackers within the same three general areas (Fig 1). The first set of sites was in high-elevation whitebark pine habitat with some subalpine fir (2659–2757 m). The second set was in mid-elevation lodgepole pine habitat with some Douglas-fir and Engelmann spruce (2187–2265 m). The third set was in mid-elevation Douglas-fir habitat with some subalpine fir, and Engelmann spruce—lodgepole pine habitat (2131–2259 m). These habitats were defined based on a simple assessment at each trapping location. I documented all conifer types visible from the location, then defined dominant trees as those composing greater than 50% of the total visible trees.

I trapped adults in mist or bow nets, using beef suet as bait, between January 28 and June 27, 2011 (*n* = 67) and January 15 and March 11, 2012 (*n* = 35). I collected body measurements and color-banded each trapped bird [26]. I attached 3.9 g (less than three percent of body weight) VHF radio transmitters (Advanced Telemetry Systems (ATS), Isanti, Minnesota, USA) with backpack harnesses to 29 and 34 of the adults in 2011 and 2012, respectively. Due to logistical constraints, I did not randomly select birds to radio-tag from among those captured. I excluded birds trapped adjacent to houses (*n* = 33), injured birds (*n* = 1; swollen foot), and birds trapped when additional radios were unavailable in the field (*n* = 5).

#### Radio-tracking

I radio-tracked Clark’s nutcrackers using homing techniques [29]. I used a digital scanning receiver (R410, ATS), and a three element folding Yagi (ATS; AF Antronics, White Heath, Illinois, USA) or H (ATS) handheld antenna. I attempted, when possible, to closely observe each radio-tagged bird for a minimum of two hours each week until the end of the field season. If I did not hear an individual’s signal, I continued to listen for it daily until I relocated the bird, visually observed that the bird was alive but had a broken antenna (i.e. the antenna had snapped and the signal was no longer being transmitted), or until the end of the field season. On eleven occasions, I attempted to relocate “missing” birds from a fixed wing aircraft using dual wing-mounted H antennas. During all observations, I documented the spatial coordinates of every location used by the bird, the microhabitat (e.g., ground, log, tree), and if in a tree, what tree species and what location in the tree (e.g., bark, foliage). I recorded its activity (e.g., foraging, flying, perching, breeding activity), the length of time it was engaged in the activity, and when possible, the food type if foraging (e.g., cached seeds, invertebrates). Unfortunately, due to logistical constraints, it was generally not possible to count the number of food items eaten during each foraging bout. I recorded locations using a portable global positioning system (GPS) unit (Garmin International Inc., Olathe, Kansas, USA).

#### Breeding behavior

To determine if breeding occurred in the population, and if so, which radio-tagged individuals bred, I observed radio-tagged and unbanded Clark’s nutcrackers throughout the prebreeding, breeding and preharvest seasons. I documented nest building, nesting behavior, and if adults were seen in the company of fledglings [26]. If a radio-tagged individual did not exhibit any nest building or nesting behavior, and was not seen in the company of fledglings, I labeled it as nonbreeding. I documented all banded and unbanded fledglings observed at all times while in the field.

### Statistical Analyses

#### Home range estimation

I collected prebreeding, breeding and preharvest location data on Clark’s nutcrackers in 2011 and 2012. I calculated area-observation curves for prebreeding through preharvest 95% fixed kernel home ranges, and the curves for a subset of ten randomly chosen individuals (for which I had a minimum of 70 points, *n* = 47) at increments of 5, up to 70 points (5 points, 10 points, etc.) [22,30]. I selected points for the area-observation curves randomly [31]. Previous simulation studies found that 30–50 points randomly drawn from multiple known distributions were sufficient to accurately define home range [32,33]. Therefore, to be conservative, I defined an individual as being adequately sampled if I obtained 30 locations, though this conventional cut-off was higher than the asymptote of the area-observation curves.

To minimize bias associated with autocorrelation, I did not use all relocation points when estimating an individual’s home range [34]. However, elimination of autocorrelation (i.e. elimination of points) might alter the apparent habitat selection patterns of the birds and alter the utilization distribution (UD) [35]. Therefore, I determined biological rather than statistical independence of points [35,36]. Biological independence is defined as the temporal interval long enough to allow an individual to move from any point within its home range to any other point within its home range [36].

To determine which points to use, I first plotted the prebreeding through preharvest season 100% minimum convex polygon (MCP) of each individual with ≥30 points. These points included each locational point once per observation regardless of how long the bird stayed at the point, and only included individuals in the first year radio-tracked. With these data, I determined the median length (the longest distance between two boundaries) of a home range. To estimate the rate a bird could travel, I quantified the rate of movement (m per min) between consecutive points during all focal observations during which the bird was continuously tracked. The maximum speed I observed an individual flying was 48 km per hr, consistent with Vander Wall et al.’s [16] estimate of 45 km per hr. If flying 48 km per hr, an individual could cross the median length of a home range in four minutes; therefore, biological independence of points was achieved during this time [29,37]. For all analyses using “points” hereafter, unless stated otherwise, I used points which were separated by at least four minutes, including the same location more than once if the individual stayed at the location for ≥4 minutes. During the nesting period, I only included the point for the first observation made at the nest, to ensure home range size estimates were not biased due to repeated observations made at the nest [38].

I estimated the breeding season home range of each individual with ≥30 points during the breeding season. One bird was tracked in both 2011 and 2012; to avoid pseudoreplication, I did not include its 2012 range in analyses. To estimate the 95% and 100% MCP home ranges, I used the “adehabitatHR” package [39] in Program R (version 3.1.0, R Development Core Team). To estimate the 50%, 95% and 99% fixed kernel breeding season home ranges, I used the Geospatial Modeling Environment (version 0.7.2.1) software [32,40,41]. I used the ‘plug-in’ method for calculating the bandwidth parameter because of better convergence and reasonable tradeoffs between bias and variance compared to the commonly used reference and least squares methods [42–44]. One individual had a bimodal range: it regularly used one area, then moved to a distant, separate area, where it remained for the rest of the breeding season. For this individual, for each of the 50%, 95% and 99% fixed kernel methods, I calculated two separate home ranges. I then added the area of each pair of home ranges together. I present the 95% fixed kernel home range sizes which I use in analyses, as well as the 50% and 99% fixed kernel and 95% and 100% MCP home range sizes to compare my home range estimates to those of other studies.

I conducted Kendall's rank correlations to ensure that I had adequately sampled individual locations during the breeding season. There was no correlation between the 95% fixed kernel home range size and the number of points per individual (*n* = 55, tau = 0.12, *P* = 0.2), or the home range size and the number of days tracked (*n* = 55, tau = 0.05, *P* = 0.6) [33,45]. I used home range sizes calculated by the 95% fixed kernel method in all statistical analyses.

To compare home range size of 2011 nonbreeders and 2012 breeders, for each method of home range estimation, I square root transformed the estimated home ranges sizes, and used a t-test. I included only one randomly selected bird from each mated pair. Due to the low sample size (*n* = 3) of 2012 nonbreeders causing unbalanced sample sizes, I did not include 2012 nonbreeders in these analyses.

#### Habitat selection

I constructed a geospatial layer of land cover types using map data from five vegetation maps (S1 Table). When discrepancies occurred, the layers were prioritized in the order listed. I classified habitat into ten categories (Table 1). The six whitebark pine health categories are those described in the whitebark pine stand-level condition assessment [46]. The ecologically-based categories were assigned based on spatial data on canopy damage and stand structure for use in prioritizing stands for protection and restoration [46]. The categories were stable through 2011 and 2012 because, though low numbers of whitebark pines continued to die, the large-scale mountain pine beetle epidemic ended at the study area due to a cold-snap in early autumn 2009 [47]. I radio-tracked and observed individuals foraging in all six available conifer habitats in the study area. When they foraged on seeds vs. alternative foods, I primarily observed Clark’s nutcrackers foraging on whitebark pine, limber pine and Douglas-fir seeds. Therefore, each of these conifers was categorized separately from all other conifers. Non-conifer habitat was included as a separate category.

**Table 1:** Habitat categories.

Habitat Categories	Average % (± SEM) of available habitat on landscape (in and within 32 km of each home range)
Whitebark pine, very low mortality, no to very low mountain pine beetle* activity	1.2 ± 0.1%
Whitebark pine, low mortality, low mountain pine beetle activity	1.8 ± 0.1%
Whitebark pine, moderate to high mortality, low to moderate mountain pine beetle activity	3.1 ± 0.2%
Whitebark pine, high mortality, very high mountain pine beetle activity	4.9 ± 0.4%
Whitebark pine, very high mortality, very low mountain pine beetle activity, all or most of whitebark pine overstory has died	0.1 ± 0.0%
Whitebark pine, burned	0.1 ± 0.0%
Limber pine	0.3 ± 0.0%
Douglas-fir	5.8 ± 0.2%
Other conifers (Engelmann spruce, lodgepole pine, and/or subalpine fir)	35.9 ± 0.5%
Non-conifer (may contain isolated trees, and isolated small stands)	47.0 ± 0.8%

* *Dendroctonus ponderosae*

I assessed Clark’s nutcracker home range and within home range habitat selection with resource selection indices [48]. In the second-order selection [49], I compared the habitat within the home range of each bird with the available habitat on the landscape. Available habitat was defined as the habitat within the home range and within 32 km of each home range boundary. I designated the buffer as 32 km because previous research documented that Clark’s nutcrackers will travel up to 32.6 km from their summer home range (which they assumed was equivalent to their breeding season home range) to harvest seeds [22]. In the third-order selection [49], I compared the proportion of habitat used within the home range (based on habitat at GPS locations where each bird was observed) with the proportion of habitat available within their home range.

For each bird, I calculated second- and third-order selection ratios for each of the ten habitat categories (*i*) as w_i_ = (proportion used habitat_i_)/(proportion available habitat_i_). To calculate a resource selection index, I then standardized: median Manly beta index (b_i_) = (selection ratio_i)_/(sum of selection ratios for all habitat types) [50]. The standardized resource selection function is the probability that for any selection event, an individual would choose habitat *i* over all others, assuming all habitats are available in equal proportion.

For both second and third-order selection, I tested habitat selection using a chi-square for each bird, with a Design III analysis in the “adehabitatHS” package [39] in Program R. I tested if overall habitat selection for each group and for each individual were significantly different from random. For the tests of group selection, to meet the assumption of independence, I removed one randomly selected individual of each mated pair from the analyses (*n* = 3 in 2011, *n* = 8 in 2012). I then determined the Bonferroni 95% confidence intervals for population selection ratios for all birds in 2011 (all nonbreeding; *n* = 22) and breeding birds in 2012 (*n* = 19). Assumptions included independence between individuals, all individuals selected habitat in a similar way though as expected there was some variation, no territoriality, and all individuals had equal access to all available resource units. Using radio-tracking to detect locations of individuals circumvented the issue of imperfect detection.

#### Foraging behavior and diet

I classified each foraging event by the food type (e.g., invertebrates, seeds retrieved from cache, Douglas-fir seeds). Due to the size variation between seeds, it was possible to determine that the seeds retrieved from caches were likely whitebark pine, and were not Douglas-fir; however, I was not able to exclude the possibility that some retrieved cached seeds were limber pine. However, limber pines in the study area were few and patchily distributed. Though they are an important late summer food source, the majority of seeds were eaten immediately rather than cached (T. D. Schaming personal observation).

I compared the proportion of the foraging observations composed of each food type between 2011 and 2012 using binomial tests. I then tested foraging habitat selection using a chi-square for each bird, with a Design III analysis in the “adehabitatHS” package [39] in Program R. I tested if overall foraging habitat selection for each group and for each individual were significantly different from random. For the tests of group selection, I removed one randomly selected individual of each mated pair from the analyses (*n* = 3 in 2011, *n* = 8 in 2012). I then determined the Bonferroni 95% confidence intervals for population selection ratios for all birds in 2011 (all nonbreeding; *n* = 22) and breeding birds in 2012 (*n* = 19). To assess if foraging habitat predicted food types eaten, I used a chi-square to determine if the food types composing >2.5% of the diet (excluding suet, which I used as bait for trapping, and unknown food types) were more likely to be eaten in specific habitats.

#### Other

I used R to perform all analyses, unless otherwise stated. I checked for normality and homogeneity of variance, applied *P ≤* 0.05 as the significance level, and reported means ± standard error.

#### Data

All of my original data from which this article is based are deposited at Figshare http://dx.doi.org/10.6084/m9.figshare.1439490. Four sets of habitat maps were obtained from third parties and are available upon request. Data from the whitebark pine stand-level condition assessment are available from The Greater Yellowstone Whitebark Pine Subcommittee (contact the current committee chair listed on http://fedgycc.org/WhitebarkPineOverview.htm). The Bridger-Teton National Forest and Grand Teton National Park maps can be obtained from Nancy Bockino (Nancy_Bockino@nps.gov, Grand Teton National Park). The Shoshone National Forest maps can be obtained from Janice Wilson (janicewilson@fs.fed.us, U.S. Forest Service Rocky Mountain Region Regional Office, Geospatial Services). Wyoming GAP analysis vegetation maps are available online from the U.S. Geological Survey National Gap Analysis Program Land Cover Data Portal (http://gapanalysis.usgs.gov/gaplandcover/).

## Results

### Home Range Estimation

In 2011 and 2012, 83% (*n* = 29) and 74% (*n* = 34) of radio-tagged Clark’s nutcrackers remained on the study area through the end of the breeding season (S2 Fig). A “missing” bird may have died, lost its antenna, permanently dispersed, or temporary emigrated (not returning to the study area until after the field season ended November 20, 2011 or October 31, 2012). I recorded adequate points to determine the breeding season home range for 55 Clark’s nutcrackers (Table 2). Observations of radio-tagged birds occurred throughout the day between 0400 and 2400 hours standard time, with the heaviest sampling between 0800 and 1600 (S2 Table).

**Table 2:** The number of points recorded for and the number of separate days I followed individual Clark’s nutcrackers.

	Prebreeding, breeding and seed preharvest (combined)		Breeding season only	
	2011	2012	2011	2012
**Mean # of points ± SEM (range)**	114 ± 9 (34–178)	98 ± 5 (30–135)	156 ± 12 (44–290)	116 ± 21 (31–207)
**Mean # of days ± SEM (range)**	18 ± 2 (7–32)	15 ± 1 (4–24)	10 ± 1 (3–15)	9 ± 1 (4–14)
**# of birds**	25	31	25	30

For the combined prebreeding, breeding and seed preharvest seasons, I include radio-tagged birds for which I recorded ≥30 points (one point per location). For the breeding season only, I include birds for which I had ≥30 independent breeding season points (≥4 minutes apart, multiple points per location possible).

**Fig 2:**
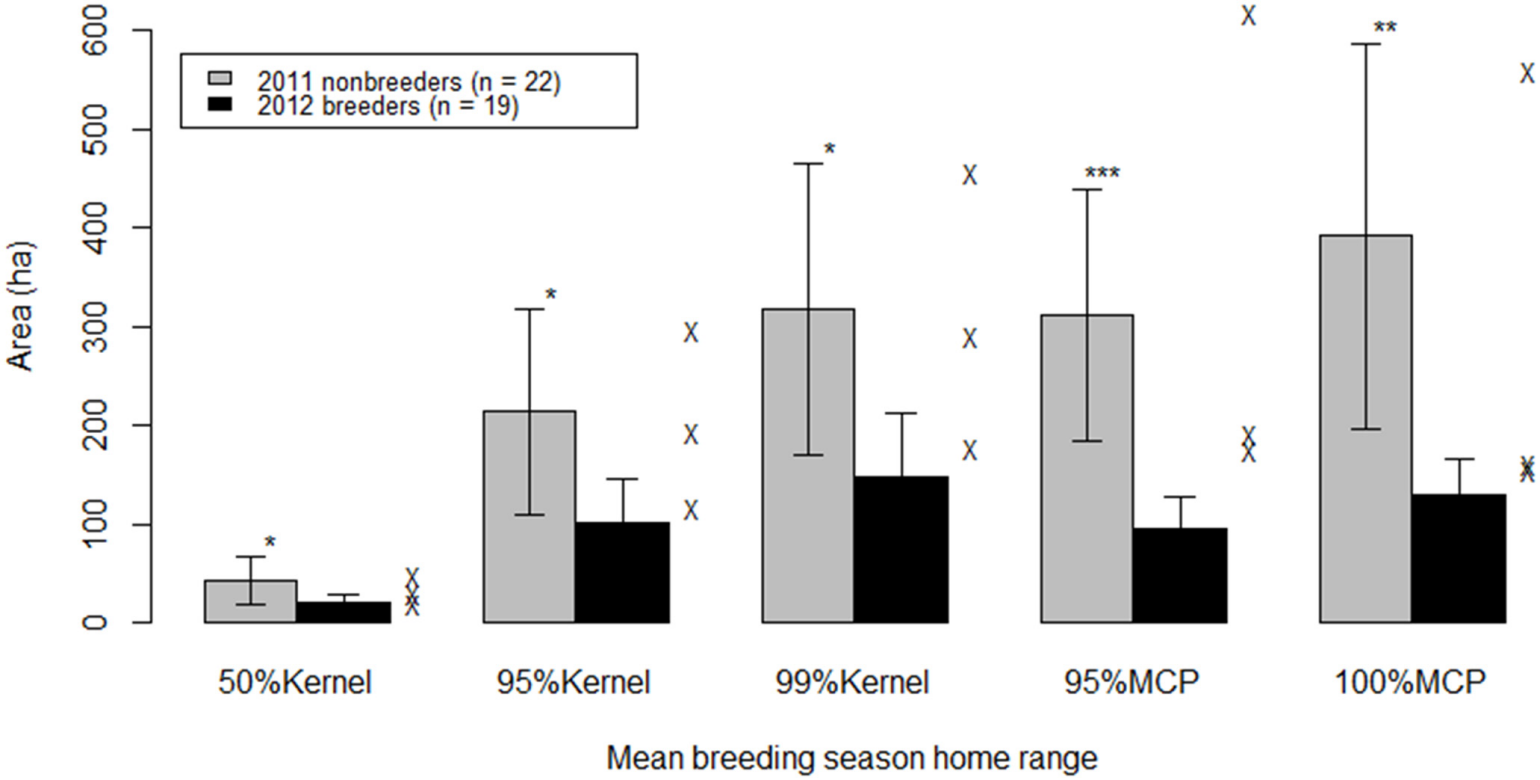
Estimated breeding season home range size. Estimated home range size of 2011 nonbreeders was significantly larger than range size of 2012 breeders. The estimated home range size for the three 2012 nonbreeders are included as X’s on the graph, but due to low sample size, were not included in analyses.

Area-observation curves reached an asymptote with an average of 26 ± 4 points (*n* = 10), consistent with Lorenz and Sullivan’s asymptote of 25 points for Clark’s nutcracker summer ranges [22]. The median length of a 100% MCP prebreeding through preharvest season home range was 3,154 m (*n* = 56, mean = 3,955, range = 864–28,141 m). Mean breeding home range size of 2011 nonbreeders was significantly larger than the range size of 2012 breeders (t = 2.4, df = 36, *P* = 0.02; Table 3 and Fig 2). Due to low sample size (*n* = 3), I did not include the 2012 nonbreeders in these analyses; however, 2012 nonbreeders’ home range sizes were more similar to 2011 nonbreeders than to 2012 breeders (Fig 2).

**Table 3:** Breeding season 95% fixed kernel home range sizes for breeding and nonbreeding birds.

Breeding status	Mean home range size ± SEM (range; ha)	
	2011	2012
Breeder	NA	101 ± 23 (15–392); n = 19
Nonbreeder	214 ± 53 (3–1231); n = 22	202 ± 53 (116–297); n = 3

### Territoriality

The Clark’s nutcracker territories overlapped considerably (S1 Text). I did not see any aggressive territorial interactions in 771.6 hours of observing radio-tagged birds in 2011 and 2012, or during numerous observations of other Clark’s nutcrackers during 1,109 person-days in the field (2009–2013). I also regularly observed all breeding and nonbreeding radio-tagged birds in flocks with >2 birds during every season.

### Habitat Selection

**Fig 3:**
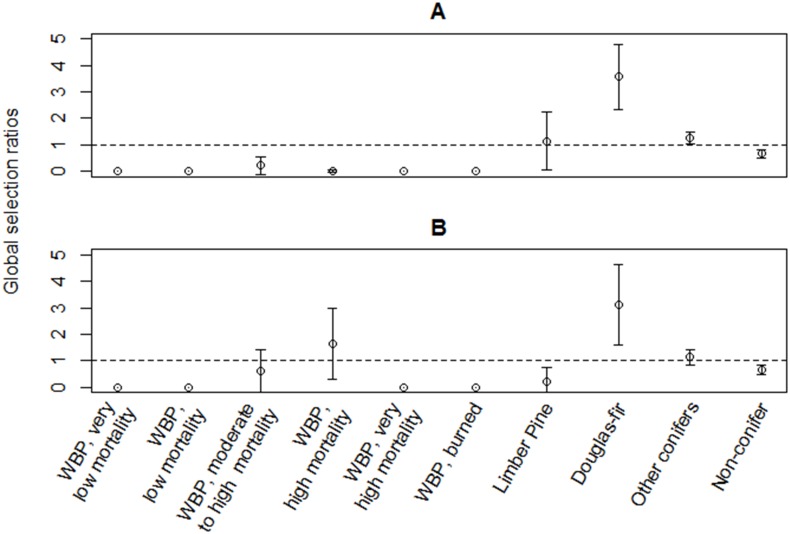
Home range habitat selection. Clark’s nutcracker selection of the home range habitat as compared to habitat available within 32 km in (**A**) 2011 (all nonbreeding birds) and (**B**) 2012 (breeding birds only). The Manly selectivity measure (± Bonferroni 95% confidence intervals (CI’s)) was used to determine if habitats were used in higher proportion than the proportion available (>1), used in the same proportion as the proportion available (CI’s include 1), used in lower proportion than the proportion available (0<X<1) or never used (0). Whitebark pine is abbreviated as WBP.

**Fig 4:**
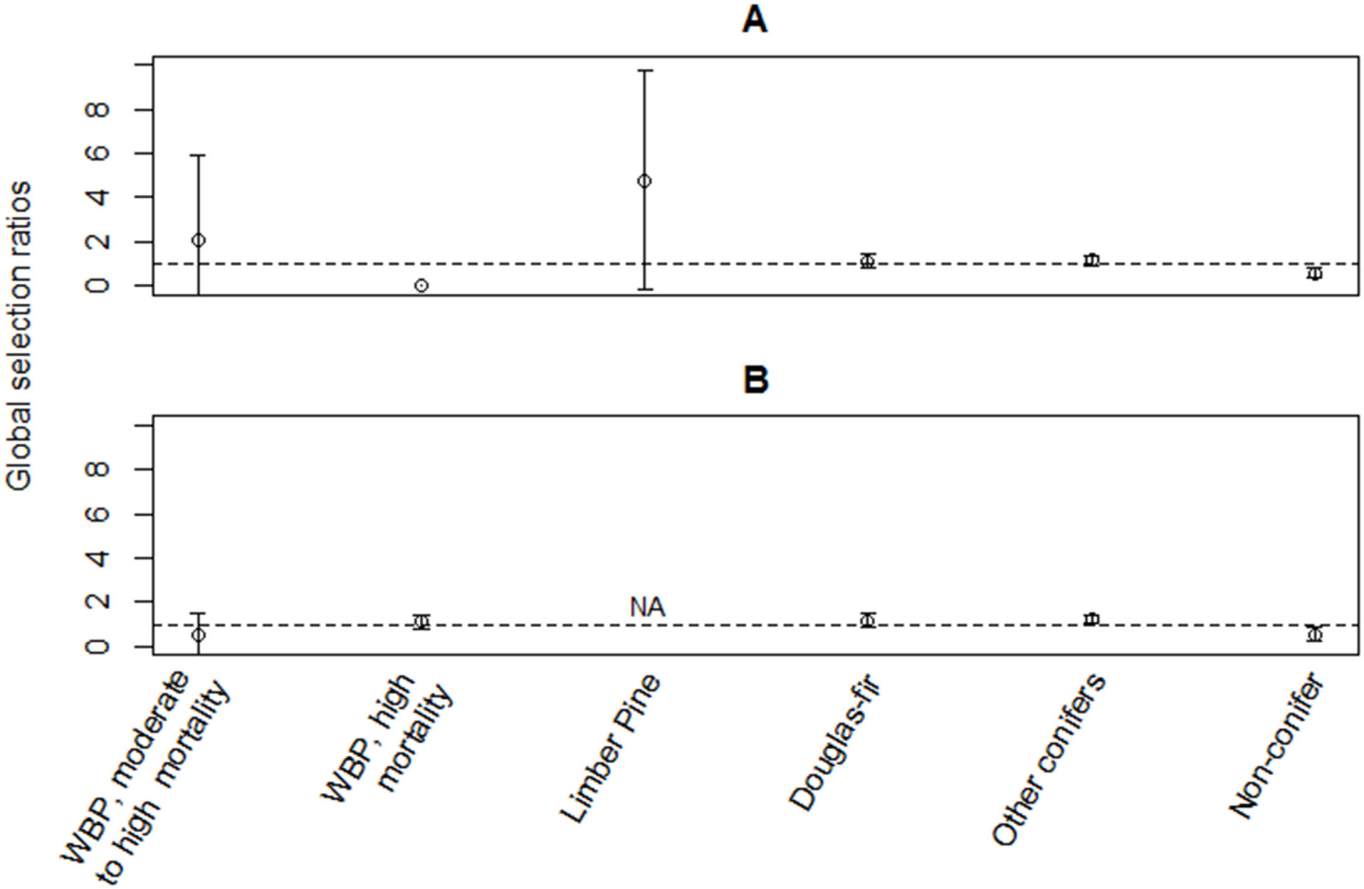
Habitat selection within the home range. Clark’s nutcracker selection of the habitat at locations from within the home range in (**A**) 2011, all nonbreeding birds and (**B**) 2012, breeding birds only. The Manly selectivity measure (± Bonferroni 95% confidence intervals (CI’s)) was used to determine if habitats were used in higher proportion than the proportion available (>1), used in the same proportion as the proportion available (CI’s include 1), used in lower proportion than the proportion available (0<X<1) or never used (0). Whitebark pine is abbreviated as WBP.

Clark’s nutcrackers in 2011 and breeding birds in 2012 did not select home range habitat randomly from within the available habitat on the landscape, or from within the home range (Table 4). In selecting home range habitat, Clark’s nutcrackers in both 2011 and 2012 only selected Douglas-fir habitat in higher proportion than the proportion available (Fig 3; S3 Table). They selected habitat without conifers in lower proportion than the proportion available, and never used whitebark pine with very low, low, or very high mortality, or burned whitebark pine. In selecting locations for all behaviors (e.g., foraging, flying, perching, breeding activity) from within the home range, Clark’s nutcrackers only showed a slight positive selection for one habitat, other conifers, in 2012 (Fig 4; S4 Table). All other available habitats were selected according to availability or in lower proportion than the proportion available. Limber pine’s large confidence intervals were due to the variability of selection between individuals: 24% of the individuals selected limber pine, whereas 76% did not use limber pine at all.

**Table 4:** Home range and within home range habitat selection.

	Habitat selection			
	Home range vs. habitat available on landscape		Locations of birds vs. habitat available in home range	
	2011	2012	2011	2012
**Random or nonrandom**	Nonrandom	Nonrandom	Nonrandom	Nonrandom
XL2²	16698967	9628923	507	348
**df**	59	53	45	44
***P***	*<* 0.001	*<* 0.001	*<* 0.001	*<* 0.001
**# of birds**	22	19	22	19
**Fig.**	3		4	

### Foraging Behavior and Diet

I observed foraging events of radio-tagged Clark’s nutcrackers 358 and 293 times during the breeding season in 2011 and 2012, respectively (Table 5). On average, I observed foraging 14 ± 2 times per individual in 2011 (*n* = 26), and 9 ± 1 times per individual in 2012 (*n* = 33). Over the 103 day breeding season each year, I observed foraging on 57 days in 2011 and 54 days in 2012. On average, I observed foraging 6 ± 1 and 5 ± 1 times per day (with foraging observations) in 2011 and 2012, respectively.

**Table 5:** Food consumed during foraging events.

Food type	# of events (%)	
	2011	2012
Seed retrieved from cache	2 (1%)	26 (9%)
Invertebrates	152 (42%)	73 (25%)
Douglas-fir cone (on ground or in tree)	26 (7%)	28 (10%)
Suet (trapping sites)	30 (8%)	7 (2%)
Limber pine cone	1 (0.3%)	2 (1%)
Engelmann spruce cone	4 (1%)	6 (2%)
Lodgepole pine cone	8 (2%)	5 (2%)
Subalpine fir cone	0 (0%)	1 (0.3%)
Douglas-fir male cone	4 (1%)	0 (0%)
Lodgepole pine male cone	0 (0%)	2 (1%)
Douglas fir buds	1 (0.3%)	0 (0%)
Dead animal	3 (1%)	3 (1%)
Rodent (depredated)	0 (0%)	1 (0.3%)
Unknown—on ground	117 (33%)	120 (41%)
Unknown—in tree	10 (3%)	19 (6%)

**Fig 5:**
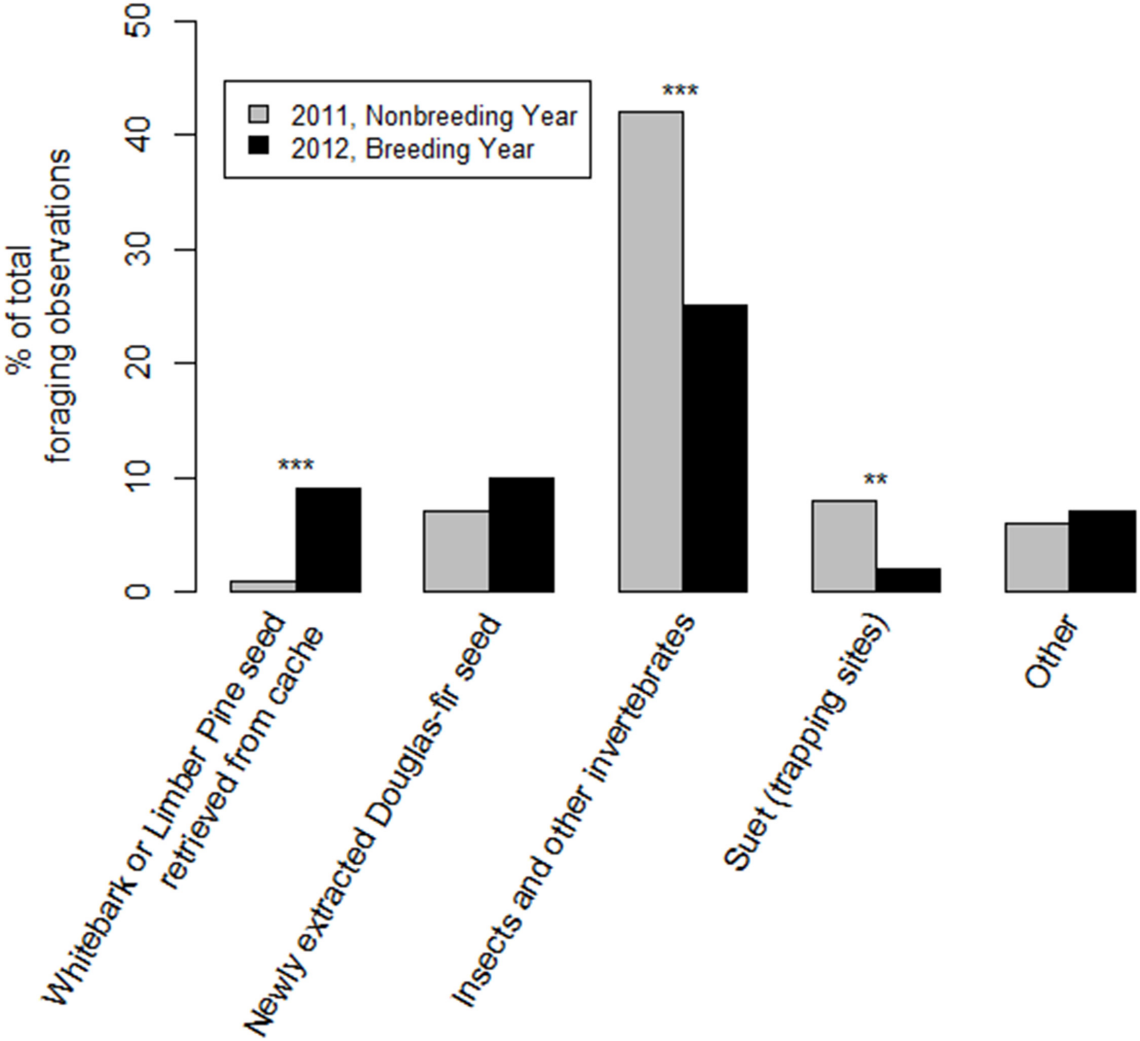
Food types. Percentage of known food types eaten during independent foraging events. Numbers do not add up to 100, as foods consumed during <2.5% of the events, and unknown food items are not included in the graph.

I observed individuals eating significantly more invertebrates (χ ^2^ = 21.2, df = 1, *P <* 0.001), and suet (χ^2^ = 9.7, df = 1, *P* = 0.002) in 2011, and significantly more seeds retrieved from caches (χ ^2^ = 25.1, df = 1, *P <* 0.001) in 2012 (Fig 5). Clark’s nutcrackers foraged on similar proportions of newly extracted Douglas-fir seeds in both years (χ ^2^ = 0.8, df = 1, *P* = 0.4). When the food type was undetermined, the majority of the time foraging occurred on the ground vs. in the trees. It is unlikely that there was a bias between years in the percentage of specific food types listed as unknown. When foraging, Clark’s nutcrackers in 2011 and breeding Clark’s nutcrackers in 2012 did not select foraging habitat at locations randomly from within the home range (*n* = 25, XL2² = 83.9, df = 39, *P <* 0.001, and *n* = 27, XL2² = 57.4, df = 32, *P* = 0.004, respectively; Table 6 and S5 Table).

**Table 6:** Clark’s nutcracker foraging habitat selection from within the home range.

Habitat	Food type						
	All		Invertebrates		Douglas-fir seeds		Seed caches
	2011	2012	2011	2012	2011	2012	2012
Whitebark pine, moderate to high mortality	0	0	0	0	0	0	0
Whitebark pine, high mortality	0	**+**	0	=	0	0	=
Limber pine	=	NA	=	NA	0	NA	NA
Douglas-fir	=	=	=	=	=	=	=
Other conifers	=	=	=	=	=	=	=
Non-conifer	=	=	=	-	=	=	=

The habitat within the home range was considered the available habitat, and the habitat where foraging events occurred was considered used habitat. “+” = habitat used in higher proportion than the proportion available; “=“ = habitat used in the same proportion as the proportion available; “-” = habitat used in lower proportion than the proportion available; “0” = habitat never used; “NA” = habitat was not available within home ranges. Habitat selection for foraging on seed caches was not included for 2011 as I only observed two events.

## Discussion

### Habitat Selection

These results suggest that, at my study site in the Greater Yellowstone Ecosystem, Douglas-fir habitat is the most important breeding season habitat for Clark’s nutcrackers. Clark’s nutcrackers selected Douglas-fir habitat in two years with contrasting food availability and breeding status. This selection occurred following a low whitebark pine cone crop, when population-wide nonbreeding occurred, and birds could therefore range more widely to track ephemeral food sources. It also occurred following a high whitebark pine cone crop when the majority of birds bred and were constrained to a nest site. It is unlikely that individuals were excluded from high quality habitat due to territoriality, because I did not observe territorial behavior, and home ranges overlapped extensively. Previous observational research had documented Clark’s nutcrackers using Douglas-fir habitat during the breeding season [11,51]. However, this is the first systematic study of space use of radio-tracked Clark’s nutcrackers to document breeding season selection of Douglas-fir habitat.

Clark’s nutcrackers specialize on large seeded pines, which are the whitebark pines and limber pines in the Greater Yellowstone Ecosystem [9,10]. I observed the majority of the radio-tagged Clark’s nutcrackers eating whitebark pine seeds between July and September in both 2011 and 2012 (T. D. Schaming personal observation). However, for their breeding season home range, the birds did not select either healthy or degraded whitebark pine, or limber pine habitats. This finding supports previous observations of infrequent use of whitebark pine communities during the breeding season [See 6]. For example, Tomback [17] observed that after whitebark pine cone crops were depleted, Clark’s nutcrackers in the Sierra Nevada migrated to lower elevations, where they stayed for the winter and spring breeding season. In contrast, in the only previous formal study of space use of individually marked Clark’s nutcrackers, Lorenz [27] determined that resident summer ranges contained proportionately more parkland whitebark pine (whitebark pine dominated, <10% canopy cover) and mixed forest compared to availability. Though she did not evaluate breeding season range separately, due to caching locations, she inferred that the summer range was also the winter and spring breeding season range.

Though Clark’s nutcrackers did not positively select moderate to high and high mortality whitebark pine habitats, selection did vary for those habitats. In 2012, the year following a high whitebark pine cone crop, Clark’s nutcrackers selected the two habitats in proportion to availability. In contrast, in 2011, the year following a low whitebark pine cone crop, they selected those habitats less than expected compared to availability. It is reasonable that Clark’s nutcrackers would use whitebark pine habitats less after a low whitebark pine crop, because a lower cone crop likely translates to fewer cached seeds. Though Clark’s nutcrackers transport their seeds up to 32.6 km, they also regularly cache seeds close to the parent trees [17,52]. The high spring snowpack in 2011, as compared to 2012, may have also contributed to the lower selection of the high elevation whitebark pine habitats in 2011. Overall, snow melted faster at lower elevations (T. D. Schaming personal observation). The finding that Clark’s nutcrackers adjusted their breeding season selection of whitebark pine habitat between years suggests that, in some years, even though it is not positively selected, whitebark pine habitat may be used more than previously expected during the spring breeding season.

Clark’s nutcrackers may have selected Douglas-fir because of its low elevation, milder winter conditions; however, limber pine and other conifer habitats (Engelmann spruce, lodgepole pine, and/or subalpine fir) were also located at lower elevations. The birds only used limber pine habitat in the proportion available in 2011, and used it less than available in 2012. Clark’s nutcrackers did select other conifer habitat following a low whitebark pine cone crop, but the selection was weak. These results suggest that for their breeding season home range, Clark’s nutcrackers are specifically selecting habitat with an available seed source, rather than just milder winter conditions.

Though it is only a sample size of two years, this variation in selection suggests that Clark’s nutcrackers alter their space use depending on demographic and/or environmental conditions, such as breeding condition or whitebark pine cone crop. Understanding the variation in size of home range and habitats selected in different years aids managers in determining the amount and diversity of habitats necessary for Clark’s nutcrackers to persist in an ecosystem.

### Foraging Behavior and Diet

When foraging within the home range, Clark’s nutcrackers selected high mortality whitebark pine habitat in 2012 in higher proportion than available. Given that past research has documented seed caching near parent trees [12], it is unsurprising that individuals are more likely to select some whitebark pine habitats for foraging in years following a large cone crop. The birds consistently selected Douglas-fir, other conifer and non-conifer habitat in proportion to availability both years. When specifically foraging for the three most common food sources (>2% of identified foraging events), invertebrates, Douglas-fir seeds and seed caches, they showed no positive selection for a specific habitat. Though Clark’s nutcrackers specialize on large seeded pines, they are opportunistic foragers [6]. Due to their varied diet, it is reasonable that the birds forage when the opportunity arises (e.g. dead animal, suet), regardless of the habitat.

Though they did not select Douglas-fir habitat for foraging disproportionate to availability, the Clark’s nutcrackers selected Douglas-fir habitat for the home range. Hence, availability of Douglas-fir habitat was already higher than expected within the home range. Therefore, foraging in Douglas-fir in proportion to availability shows strong selection of Douglas-fir habitat. The stability of selection of Douglas-fir habitat across years with variable demographic and environmental conditions validates its importance as foraging habitat. On the other hand, the Douglas-fir cone crop was high each year, 2008–2014 (T. D. Schaming personal observation); therefore, it is unclear how Clark’s nutcracker habitat selection would change in years with a low Douglas-fir cone crop.

Both years, I observed individuals foraging on newly extracted Douglas-fir seeds in Douglas-fir, other conifer and non-conifer habitats. Douglas-fir seeds were therefore available in multiple habitats, not just the habitat dominated by Douglas-fir stands. Foraging on Douglas-fir in all three habitat types emphasizes the importance of Douglas-fir as a food source. The inclusion of Douglas-fir seeds in the spring diet was previously documented by Giuntoli and Mewaldt’s [23] analysis of Clark’s nutcracker stomach contents. However, it is unclear whether the seeds were newly harvested or cached the previous autumn. Clark’s nutcrackers have been observed to eat seeds which remained in cones through the spring: Tomback documented Clark’s nutcrackers feeding on Jeffrey pine cones during the breeding season [53]. Habitats which contain seeds remaining available through the spring may be particularly important for Clark’s nutcrackers in locations with declining whitebark pine ecosystems.

In contrast to the stability of the importance of Douglas-fir seeds, I observed Clark’s nutcrackers foraging on few seed caches, even after a high whitebark pine cone crop (S2 Text). Though it is possible that some of the unknown foraging events included seed caches, it is unlikely that I was unable to detect seed cache retrieval in most situations. Past research documented Clark’s nutcrackers eating and feeding nestlings cached seeds in the spring [11,23,53]. However, my results suggest that the importance of cached seeds in the breeding season diet may be overestimated. Alternatively, it may be highly variable between regions. Even in 2012, seed caches accounted for only 9% of the breeding season foraging events. Douglas-fir cones accounted for a similar 10%, while invertebrates were eaten in approximately three times the number of foraging events (42% in 2011; 25% in 2012). Similarly, previous research found that during the breeding season, 44–100% of Clark’s nutcracker stomach contents contained arthropods [23]. Invertebrates may be a more important part of the breeding season diet, at least in some areas, than previous research suggested [11].

### Conservation Implications

Whitebark pines are declining in the Greater Yellowstone Ecosystem [13,54]. Land managers have focused on restoring whitebark pine habitat, with the assumption that Clark’s nutcrackers will be available to resume seed dispersal [21]. They presume the birds will disperse seeds once the whitebark pine forests reach an adequate state of health [21]. This, however, assumes that Clark’s nutcrackers will persist in, or move back into locations once whitebark pine habitats are restored.

The Clark’s nutcracker is a partially migratory, irruptive seed specialist [16]. Dohms and Burg [55] suggested there are high levels of gene flow among populations, unrestricted by potential barriers such as mountain ranges. Therefore, it is possible that Clark’s nutcrackers may decline or become extinct locally, but could then recolonize an area once habitat improves, providing they survive elsewhere. However, given the widespread nature of the decline of five-needle pines, the best management practice may be to ensure a stable population of Clark’s nutcrackers persists in the ecosystem.

It is important to consider which measures could maintain viable Clark’s nutcracker populations. Lorenz suggested that increasing the health of ponderosa pine stands in her study area in the Cascade Range may sustain Clark’s nutcracker populations during whitebark pine recovery [27]. This seems straightforward, as every individual in her study harvested and cached ponderosa pine seeds in the autumn. Even when whitebark pine seeds were available, not all birds harvested whitebark pine seeds. Unlike ponderosa pine, Douglas-fir are unlikely to replace whitebark pine in the diet due to their lower nutritional value and longer handling time [26]. Nevertheless, in my study area, Clark’s nutcrackers selected Douglas-fir for their breeding season home range. This selection has important implications for habitat conservation planning. Though they may not be able to persist solely on Douglas-fir seeds, the foraging provided by Douglas-fir stands may provide a critical alternative seed source in the Greater Yellowstone Ecosystem, helping the Clark’s nutcrackers to meet their foraging requirements.

To my knowledge, whitebark pine restoration strategies focus nearly exclusively on whitebark pine forests. Managers do not account for the mobility of Clark’s nutcracker populations. Instead of managing whitebark pine in isolation, they may need to consider the different habitats Clark’s nutcrackers use throughout the year, as well as the variability of those habitats in years with differing demographic and environmental conditions.

The results of this study may be more representative of Clark’s nutcracker behavior in degraded whitebark pine habitat, rather than healthy forest communities. However, the importance of alternative seed sources, such as Douglas-fir, may be particularly critical in these degraded habitats. With the widespread decline of their primary food sources, five-needle white pines, and in particular whitebark pine, habitats with alternative food sources may be increasingly important for supporting Clark’s nutcracker populations. Due to the reduction in primary habitat, these habitats may offer refugia and may be critical for long-term population viability [56,57]. Optimizing landscape level management of whitebark pine restoration may be critical to conserving whitebark pine communities in the Greater Yellowstone Ecosystem. I specifically suggest that managers consider restoration locations adjacent to a mosaic of habitats which specifically includes Douglas-fir. By extrapolation, whitebark pine restoration efforts in other regions may consider prioritizing restoration of whitebark pine stands near alternative seed sources.

Managing wide-ranging species that require seasonally distinct and spatially discrete habitats can be challenging [58]. Nevertheless, traditional approaches of focusing on protection of primary habitat may need to be reassessed in the face of a changing climate and widespread habitat decline [59]. Despite the constraints, policy makers may need to consider protecting broader areas to encompass all the resource requirements of populations [60].

## Supporting Information

S1 Fig Timeline of Clark’s nutcracker annual cycle in 2011 and 2012. (The focus of this study is on the breeding season.)
(TIF)

S2 Fig Fate of radio-tagged Clark’s nutcrackers during breeding and preharvest seasons. (TIF)

S1 Table Vegetation maps used to create the geospatial layer of land cover types. (DOCX)

S2 Table Time of day during which I observed radio-tagged Clark’s nutcrackers. (DOCX)

S3 Table The Manly selectivity measure (± Bonferroni 95% confidence intervals (CI’s)) used to evaluate Clark’s nutcracker selection of the home range habitat as compared to habitat available within 32 km. (DOCX)

S4 Table The Manly selectivity measure (± Bonferroni 95% confidence intervals (CI’s)) used to evaluate Clark’s nutcracker selection of the habitat at locations from within the home range. (DOCX)

S5 Table The Manly selectivity measure (± Bonferroni 95% confidence intervals (CI’s)) used to evaluate Clark’s nutcracker selection of foraging habitat. (DOCX)

S1 Text Home range overlap. (DOCX)

S2 Text Variation in foraging on seed caches between years. (DOCX)

## References

[1] Caro T. Behavioral Ecology and Conservation Biology. Oxford University Press, USA; 1998.

[2] Runge CA, Tulloch A, Hammill E, Possingham HP, Fuller RA. Geographic range size and extinction risk assessment in nomadic species: Geographic Range Dynamics of Nomadic Birds. Conserv Biol. 2015;29: 865–876. 10.1111/cobi.12440 25580637 PMC4681363

[3] Sherry TW, Holmes RT. Winter Habitat Quality, Population Limitation, and Conservation of Neotropical-Nearctic Migrant Birds. Ecology. 1996;77: 36.

[4] Faccio SD. Postbreeding Emigration and Habitat Use by Jefferson and Spotted Salamanders in Vermont. J Herpetol. 2003;37: 479–489. 10.1670/155-02A

[5] Tomback DF, Achuff P. Blister rust and western forest biodiversity: ecology, values and outlook for white pines: Blister rust and western forest biodiversity. For Pathol. 2010;40: 186–225. 10.1111/j.1439-0329.2010.00655.x

[6] Tomback DF. Clark’s Nutcracker (Nucifraga columbiana) In: Poole A, editor. The Birds of North America Online. Ithaca, New York: Cornell Lab of Ornithology; 1998 Available: http://bna.birds.cornell.edu/bna/species/331. Accessed 11 June 2014.

[7] McKinney ST, Fiedler CE, Tomback DF. Invasive pathogen threatens bird-pine mutualism: implications for sustaining a high-elevation ecosystem. Ecol Appl. 2009;19: 597–607. 19425424 10.1890/08-0151.1

[8] Barringer LE, Tomback DF, Wunder MB, McKinney ST. Whitebark Pine Stand Condition, Tree Abundance, and Cone Production as Predictors of Visitation by Clark’s Nutcracker. Newsom LA, editor. PLoS ONE. 2012;7: e37663 10.1371/journal.pone.0037663 22662186 PMC3360761

[9] Hutchins HE, Lanner RM. The central role of Clark’s nutcracker in the dispersal and establishment of whitebark pine. Oecologia. 1982;55: 192–201. 28311233 10.1007/BF00384487

[10] Tomback DF. Dispersal of whitebark pine seeds by Clark’s nutcracker: a mutualism hypothesis. J Anim Ecol. 1982;51: 451–467.

[11] Mewaldt LR. Nesting behavior of the Clark Nutcracker. The Condor. 1956;58: 3–23.

[12] Vander Wall SB. Foraging of Clark’s Nutcrackers on rapidly changing pine seed resources. Condor. 1988;90: 621–631.

[13] Tomback DF, Arno SF, Keane RE. Whitebark pine communities: ecology and restoration. Washington D.C. USA: Island Press; 2001.

[14] Tomback DF, Linhart YB. The evolution of bird-dispersed pines. Evol Ecol. 1990;4: 185–219.

[15] Davis J, Williams L. Irruptions of the Clark nutcracker in California. The Condor. 1957;59: 297–307.

[16] Vander Wall SB, Hoffman SW, Potts WK. Emigration behavior of Clark’s Nutcracker. The Condor. 1981;83: 162–170.

[17] Tomback DF. Foraging strategies of Clark’s Nutcracker. Living Bird. 1978;16: 123–160.

[18] Farnes PE. Snowtel and snow course data: describing the hydrology of whitebark pine ecosystems. In: Schmidt, WC and MacDonald KJ, compilers. Proceedings—Symposium on Whitebark Pine Ecosystems: Ecology and Management of a High-Mountain Resource. Bozeman, Montana, USA 29–31 March 1989; 1990. pp. 302–304.

[19] Lanner RM. Made for each other, a symbiosis of birds and pines. New York, New York, USA: Oxford University Press; 1996.

[20] Ellison AM, Bank MS, Clinton BD, Colburn EA, Elliott K, Ford CR, et al Loss of foundation species: consequences for the structure and dynamics of forested ecosystems. Front Ecol Environ. 2005;3: 479–486.

[21] Keane RE, Tomback DF, Aubry CA, Bower AD, Campbell EM, Cripps CL, et al A range-wide restoration strategy for whitebark pine (Pinus albicaulis) Gen. Tech. Rep. RMRS-GTR-279. Fort Collins, CO: U.S Department of Agriculture, Forest Service, Rocky Mountain Research Station; 2012 108 p.

[22] Lorenz TJ, Sullivan KA. Seasonal Differences in Space Use by Clark’s Nutcrackers in the Cascade Range. The Condor. 2009;111: 326–340.

[23] Giuntoli M, Mewaldt LR. Stomach contents of Clark’s nutcrackers collected in western Montana. The Auk. 1978;95: 595–598.

[24] Vander Wall SB, Balda RP. Coadaptations of the Clark’s nutcracker and the pinon pine for efficient seed harvest and dispersal. Ecol Monogr. 1977;47: 89–111.

[25] Vander Wall SB, Hutchins HE. Dependence of Clark’s Nutcracker, Nucifraga columbiana, on conifer seeds during the postfledging period. Can Field-Nat. 1983;97: 208–214.

[26] Schaming TD. Population-Wide Failure to Breed in the Clark’s Nutcracker (Nucifraga columbiana). PLoS ONE. 2015;10: e0123917 10.1371/journal.pone.0123917 25970294 PMC4430254

[27] Lorenz TJ. Final Report: Clark's nutcracker habitat use and relative abundance in the Cascade Range. Prepared for the Seattle City Light Wildlife Research Program. Report on file with: U.S. Department of Agriculture, Forest Service, Rocky Mountain Research Station, Fire Sciences Laboratory, Missoula, Montana. Seattle City Light Wildlife Research Program. 2009.

[28] Macfarlane WW, Logan JA, Kern WR. An innovative aerial assessment of Greater Yellowstone Ecosystem mountain pine beetle-caused whitebark pine mortality. Ecol Appl. 2013;23: 421–437. 23634592 10.1890/11-1982.1

[29] White GC, Garrott RA. Analysis of wildlife radio-tracking data. Elsevier; 1990.

[30] Odum EP, Kuenzler EJ. Measurement of territory and home range size in birds. The Auk. 1955;72: 128–137.

[31] Harris S, Cresswell WJ, Forde PG, Trewhella WJ, Woollard T, Wray S. Home-range analysis using radio-tracking data—a review of problems and techniques particularly as applied to the study of mammals. Mammal Rev. 1990;20: 97–123.

[32] Seaman DE, Millspaugh JJ, Kernohan BJ, Brundige GC, Raedeke KJ, Gitzen RA. Effects of Sample Size on Kernel Home Range Estimates. J Wildl Manag. 1999;63: 739–747.

[33] Marzluff JM, Millspaugh JJ, Hurvitz P, Handcock MS. Relating resources to a probabilistic measure of space use: forest fragments and Steller’s jays. Ecology. 2004;85: 1411–1427.

[34] Kernohan BJ, Gitzen RA, Millspaugh JJ. Analysis of animal space use and movements In: Millspaugh JJ, Marluff JM, editors. Radio Tracking Animal Populations. Academic Press: San Diego, CA, USA; 2001 pp. 125–166.

[35] Barg JJ, Jones J, Robertson RJ. Describing breeding territories of migratory passerines: suggestions for sampling, choice of estimator, and delineation of core areas. J Anim Ecol. 2005;74: 139–149.

[36] Lair H. Estimating the location of the focal center in red squirrel home ranges. Ecology. 1987; 1092–1101.

[37] Otis DL, White GC. Autocorrelation of location estimates and the analysis of radiotracking data. J Wildl Manag. 1999;63: 1039–1044.

[38] Rota CT, Rumble MA, Millspaugh JJ, Lehman CP, Kesler DC. Space-use and habitat associations of Black-backed Woodpeckers (Picoides arcticus) occupying recently disturbed forests in the Black Hills, South Dakota. For Ecol Manag. 2014;313: 161–168.

[39] Calenge C. The package adehabitat for the R software: a tool for the analysis of space and habitat use by animals. Ecol Model. 2006;197: 516–519.

[40] Worton BJ. Kernel methods for estimating the utilization distribution in home-range studies. Ecology. 1989;70: 164–168.

[41] Kie JG, Baldwin JA, Evans CJ. CALHOME: a program for estimating animal home ranges. Wildl Soc Bull. 1996;24: 342–344.

[42] Jones MC, Marron JS, Sheather SJ. A brief survey of bandwidth selection for density estimation. J Am Stat Assoc. 1996;91: 401–407.

[43] Duong T, Hazelton M. Plug-in bandwidth matrices for bivariate kernel density estimation. J Nonparametric Stat. 2003;15: 17–30.

[44] Millspaugh JJ, Nielson RM, McDonald L, Marzluff JM, Gitzen RA, Rittenhouse CD, et al Analysis of resource selection using utilization distributions. J Wildl Manag. 2006;70: 384–395.

[45] Boal CW, Andersen DE, Kennedy PL. Home range and residency status of northern goshawks breeding in Minnesota. The Condor. 2003;105: 811–816.

[46] Greater Yellowstone Coordinating Committee Whitebark Pine Subcommittee. Whitebark Pine Strategy for the Greater Yellowstone Area. 2011. 41 p. Available: http://fedgycc.org/documents/WBPStrategyFINAL5.31.11.pdf. Accessed 22 October 2014. 2011.

[47] Dooley EM. Mountain pine beetle outbreaks in high elevation whitebark pine forests: The effects of tree host species and blister rust infection severity on beetle productivity. M.S. Thesis. Syracuse University. Syracuse, NY, USA; 2012.

[48] Savage RE. The relation between the feeding of the herring off the east coast of England and the plankton of the surrounding waters. Fish Investig Lond. 1931; Series 2. 12: 88 pp.

[49] Johnson DH. The comparison of usage and availability measurements for evaluating resource preference. Ecology. 1980;61: 65–71.

[50] Manly BF, McDonald L, Thomas D. Resource selection by animals: statistical design and analysis for field studies. Springer Science & Business Media; 1993.

[51] Bradbury WC. Notes on the nesting habits of the Clarke nutcracker in Colorado. The Condor. 1917;19: 149–155.

[52] Lorenz TJ, Sullivan KA, Bakian AV, Aubry CA. Cache-site selection in Clark’s Nutcracker (Nucifraga columbiana). The Auk. 2011;128: 237–247.

[53] Tomback DF. The behavioral ecology of the Clark’s Nutcracker (Nucifraga columbiana) in the eastern Sierra Nevada. Ph.D. dissertation, University of California, Santa Barbara, CA. 1977;

[54] Macfarlane WW, Logan JA, Kern WR. Using the Landscape Assessment System (LAS) to Assess Mountain Pine Beetle-Caused Mortality of Whitebark Pine, Greater Yellowstone Ecosystem, 2009: Project Report. Prepared for the Greater Yellowstone Coordinating Committee, Whitebark Pine Subcommittee, Jackson, Wyoming. Available: http://docs.nrdc.org/land/files/lan_10072101a.pdf. Accessed 18 June 2014. 2010.

[55] Dohms KM, Burg TM. Molecular markers reveal limited population genetic structure in a North American corvid, Clark’s Nutcracker (Nucifraga columbiana). PloS One. 2013;8: e79621 10.1371/journal.pone.0079621 24223982 PMC3817134

[56] Nielsen SE, Stenhouse GB, Boyce MS. A habitat-based framework for grizzly bear conservation in Alberta. Biol Conserv. 2006;130: 217–229.

[57] Aldridge CL, Boyce MS. Linking occurrence and fitness to persistence: habitat-based approach for endangered greater sage-grouse. Ecol Appl. 2007;17: 508–526. 17489256 10.1890/05-1871

[58] Baldwin RF, Calhoun AJ, deMaynadier PG. Conservation planning for amphibian species with complex habitat requirements: a case study using movements and habitat selection of the wood frog Rana sylvatica. J Herpetol. 2006;40: 442–453.

[59] Fogg AM, Roberts LJ, Burnett RD. Occurrence patterns of Black-backed Woodpeckers in green forest of the Sierra Nevada Mountains, California, USA. Article 3. Avian Conserv Ecol. 2014;9 Available: http://www.ace-eco.org/vol9/iss2/art3/ACE-ECO-2014-671.pdf.

[60] Law BS, Dickman CR. The use of habitat mosaics by terrestrial vertebrate fauna: implications for conservation and management. Biodivers Conserv. 1998;7: 323–333.

