# Supplementary figures and images for: Clark’s Nutcracker Breeding Season Space Use and Foraging Behavior
Source: PLoS One. 2016 Feb 16;11(2):e0149116. doi: 10.1371/journal.pone.0149116 (PMC4755556; doi:10.1371/journal.pone.0149116)

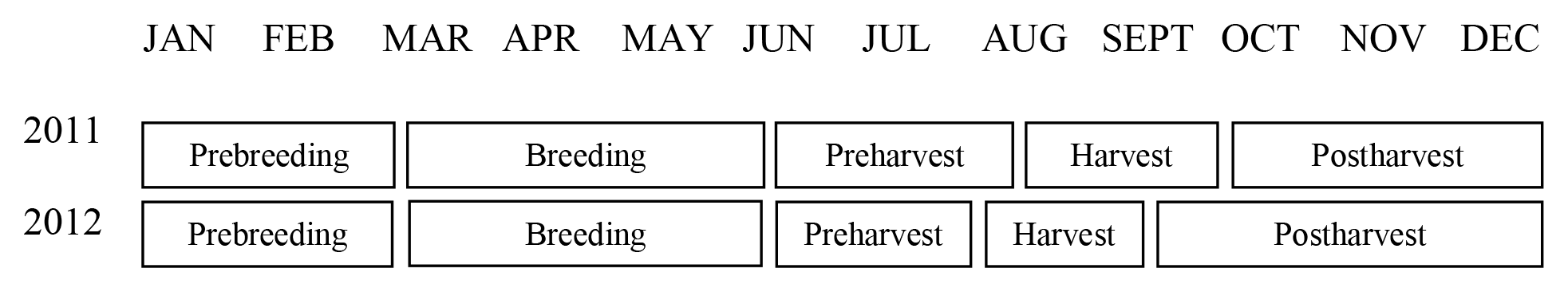

Supplement: S1 Fig — (The focus of this study is on the breeding season.) (TIF) [file pone.0149116.s001.tif]

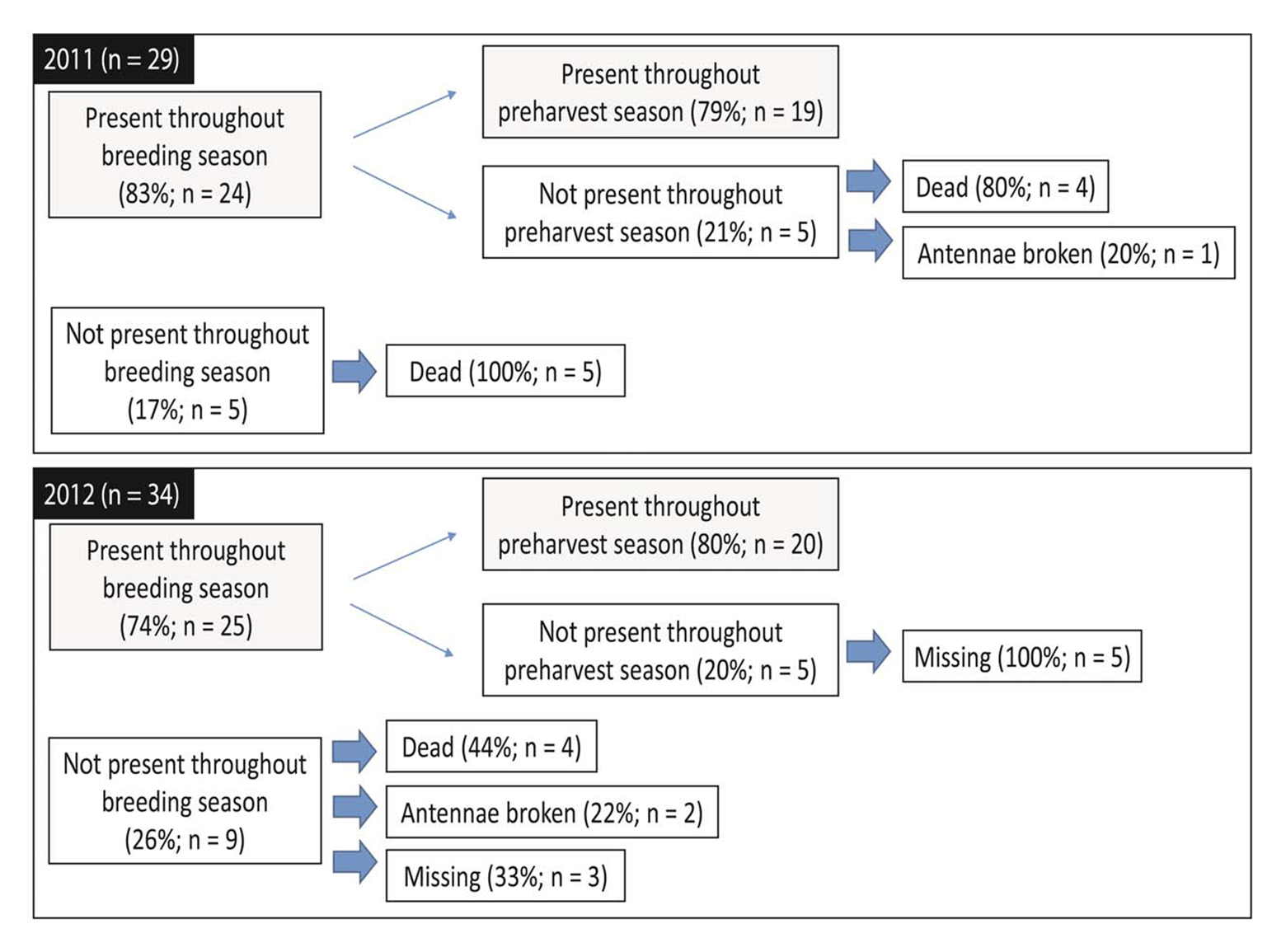

Supplement: S2 Fig — (TIF) [file pone.0149116.s002.tif]
