# Supplementary material for: Clark’s Nutcracker Breeding Season Space Use and Foraging Behavior
Source: PLoS One. 2016 Feb 16;11(2):e0149116. doi: 10.1371/journal.pone.0149116 (PMC4755556; doi:10.1371/journal.pone.0149116)
Supplement: S1 Table — (DOCX) [file pone.0149116.s003.docx]

**S1 Table. Vegetation maps used to create the geospatial layer of land cover types.**

| **Vegetation map data** | **Name of data file** | **Obtained from** |
| --- | --- | --- |
| Whitebark pine stand-level condition assessment | [1] | The Greater Yellowstone Whitebark Pine Subcommittee |
| Bridger-Teton National Forest | existveg_2007, USDA National Forest Service Remote Sensing Applications Center | Grand Teton National Park |
| Shoshone National Forest | FSVeg Spatial database, extracted March 22, 2012 | U.S. Forest Service Rocky Mountain Region (R2) Regional Office, Geospatial Services |
| Grand Teton National Park | 2005 vegetation mapping report | Grand Teton National Park |
| Wyoming GAP analysis | [2] | Online |

**References**

1. Greater Yellowstone Coordinating Committee Whitebark Pine Subcommittee. Whitebark Pine Strategy for the Greater Yellowstone Area. 2011.41 p. Available: http://fedgycc.org/documents/WBPStrategyFINAL5.31.11.pdf. Accessed 22 October 2014.

2. USGS. U.S. Geological Survey National Gap Analysis Program (GAP) Land Cover Data Portal v2.2. n.d. http://gapanalysis.usgs.gov/gaplandcover/. Accessed 26 August 2014.
