# Supplementary material for: Clark’s Nutcracker Breeding Season Space Use and Foraging Behavior
Source: PLoS One. 2016 Feb 16;11(2):e0149116. doi: 10.1371/journal.pone.0149116 (PMC4755556; doi:10.1371/journal.pone.0149116)
Supplement: S2 Table — (DOCX) [file pone.0149116.s004.docx]

**S2 Table. Time of day during which I observed radio-tagged Clark’s nutcrackers.**

| **Time** | **2011** | **2012** |
| --- | --- | --- |
| 0400-0600 | 1% | 0% |
| 0600-0800 | 4% | 1% |
| 0800-1000 | 21% | 14% |
| 1000-1200 | 29% | 28% |
| 1200-1400 | 26% | 34% |
| 1400-1600 | 18% | 21% |
| 1600-1800 | 2% | 1% |
| 1800-2000 | 0.4% | 0% |
| 2000-2200 | 0.4% | 0% |
| 2200-2400 | 0.05% | 0% |
