# Supplementary material for: Clark’s Nutcracker Breeding Season Space Use and Foraging Behavior
Source: PLoS One. 2016 Feb 16;11(2):e0149116. doi: 10.1371/journal.pone.0149116 (PMC4755556; doi:10.1371/journal.pone.0149116)
Supplement: S3 Table — (DOCX) [file pone.0149116.s005.docx]

**S3 Table. The Manly selectivity measure (± Bonferroni 95% confidence intervals (CI’s)) used to evaluate** **Clark’s nutcracker selection of the home range habitat as compared to habitat available within 32 km.**

| **Year** | **2011** | | | | **2012** | | | |
| --- | --- | --- | --- | --- | --- | --- | --- | --- |
| **Habitat** | **w_i_** | **SEM** | **CI (low)** | **CI (high)** | **w_i_** | **SEM** | **CI (low)** | **CI (high)** |
| Whitebark pine, very low mortality | 0.00 | 0.00 | 0.00 | 0.00 | 0.00 | 0.00 | 0.00 | 0.00 |
| Whitebark pine, low mortality | 0.00 | 0.00 | 0.00 | 0.00 | 0.00 | 0.00 | 0.00 | 0.00 |
| Whitebark pine, moderate to high mortality | 0.22 | 0.12 | -0.13 | 0.56 | 0.61 | 0.29 | -0.21 | 1.43 |
| Whitebark pine, high mortality | 0.01 | 0.01 | -0.02 | 0.04 | 1.64 | 0.48 | 0.29 | 2.99 |
| Whitebark pine, very high mortality | 0.00 | 0.00 | 0.00 | 0.00 | 0.00 | 0.00 | 0.00 | 0.00 |
| Whitebark pine, burned | 0.00 | 0.00 | 0.00 | 0.00 | 0.00 | 0.00 | 0.00 | 0.00 |
| Limber pine | 1.14 | 0.39 | 0.05 | 2.23 | 0.21 | 0.20 | -0.34 | 0.76 |
| Douglas-fir | 3.57 | 0.44 | 2.35 | 4.80 | 3.11 | 0.54 | 1.59 | 4.62 |
| Other conifers | 1.25 | 0.08 | 1.04 | 1.47 | 1.13 | 0.11 | 0.83 | 1.43 |
| Non-conifer | 0.67 | 0.06 | 0.51 | 0.83 | 0.66 | 0.06 | 0.49 | 0.83 |
