# Supplementary material for: Clark’s Nutcracker Breeding Season Space Use and Foraging Behavior
Source: PLoS One. 2016 Feb 16;11(2):e0149116. doi: 10.1371/journal.pone.0149116 (PMC4755556; doi:10.1371/journal.pone.0149116)
Supplement: S4 Table — (DOCX) [file pone.0149116.s006.docx]

**S4 Table. The Manly selectivity measure (± Bonferroni 95% confidence intervals (CI’s)) used to evaluate** **Clark’s nutcracker selection of the habitat at locations from within the home range.**

| **Year** | **2011** | | | | **2012** | | | |
| --- | --- | --- | --- | --- | --- | --- | --- | --- |
| **Habitat** | **w_i_** | **SEM** | **CI (low)** | **CI (high)** | **w_i_** | **SEM** | **CI (low)** | **CI (high)** |
| Whitebark pine, moderate to high mortality | 2.04 | 1.46 | -1.80 | 5.88 | 0.53 | 0.37 | -0.44 | 1.50 |
| Whitebark pine, high mortality | 0.00 | 0.00 | 0.00 | 0.00 | 1.10 | 0.13 | 0.76 | 1.44 |
| Limber pine | 4.77 | 1.88 | -0.18 | 9.72 | NA | NA | NA | NA |
| Douglas-fir | 1.14 | 0.10 | 0.88 | 1.41 | 1.15 | 0.12 | 0.84 | 1.47 |
| Other conifers | 1.14 | 0.08 | 0.94 | 1.35 | 1.23 | 0.06 | 1.06 | 1.40 |
| Non-conifer | 0.59 | 0.08 | 0.37 | 0.82 | 0.55 | 0.11 | 0.26 | 0.84 |
