# Supplementary material for: Clark’s Nutcracker Breeding Season Space Use and Foraging Behavior
Source: PLoS One. 2016 Feb 16;11(2):e0149116. doi: 10.1371/journal.pone.0149116 (PMC4755556; doi:10.1371/journal.pone.0149116)
Supplement: S5 Table — (DOCX) [file pone.0149116.s007.docx]

**S5 Table. The Manly selectivity measure (± Bonferroni 95% confidence intervals (CI’s)) used to evaluate** **Clark’s nutcracker selection of foraging habitat.**

| **Habitat** | **w_i_ ± SEM (CI)** | | | | | | |
| --- | --- | --- | --- | --- | --- | --- | --- |
|  | **All Food** | | **Invertebrates** | | **Douglas-fir seeds** | | **Seed caches** |
|  | **2011** | **2012** | **2011** | **2012** | **2011** | **2012** | **2012** |
| Whitebark pine, moderate to high mortality | never used | never used | never used | never used | never used | never used | never used |
| Whitebark pine, high mortality | never used | 1.2 ± 0.06 (1.04-1.36) | never used | 1.54 ± 0.35 (0.67-2.42) | never used | never used | 0.53 ± 0.48 (-0.66-1.72) |
| Limber pine | 4.9 ± 2.14 (-0.45-10.24) | NA | 5.15 ± 3.49 (-3.57-13.86) | NA | never used | NA | NA |
| Douglas-fir | 0.92 ± 0.11 (0.64-1.19) | 1.27 ± 0.21 (0.74-1.8) | 1.02 ± 0.18 (0.58-1.47) | 0.84 ± 0.27 (0.17-1.51) | 1.25 ± 0.26 (0.62-1.88) | 0.84 ± 0.3 (0.12-1.56) | 1.2 ± 0.56 (-0.19-2.59) |
| Other conifers | 1.28 ± 0.12 (0.97-1.58) | 1.04 ± 0.12 (0.75-1.33) | 1.21 ± 0.11 (0.93-1.49) | 1.34 ± 0.43 (0.27-2.4) | 1.19 ± 0.51  (-0.04-2.42) | 1.1 ± 0.2 (0.61-1.58) | 1.16 ± 0.24 (0.55-1.77) |
| Non-conifer | 0.67 ± 0.18 (0.22-1.11) | 0.68 ± 0.17 (0.25-1.1) | 0.64 ± 0.17 (0.21-1.08) | 0.56 ± 0.17 (0.13-1) | 0.57 ± 0.39  (-0.36-1.49) | 1.01 ± 0.48 (-0.14-2.16) | 0.8 ± 0.37 (-0.13-1.73) |
