# Supplementary material for: Clark’s Nutcracker Breeding Season Space Use and Foraging Behavior
Source: PLoS One. 2016 Feb 16;11(2):e0149116. doi: 10.1371/journal.pone.0149116 (PMC4755556; doi:10.1371/journal.pone.0149116)
Supplement: S1 Text — (DOCX) [file pone.0149116.s008.docx]

**Home range overlap**

I calculated the overlap of each bird’s breeding season home range with every other individual’s home range by superimposing each pair of two-dimensional kernels [1]. Then, I used a Mann-Whitney-Wilcoxon Test to compare the mean overlap between mates vs. the mean overlap between unmated individuals. One individual had adequate locational points to determine the breeding season home range in both 2011 and 2012. I calculated the overlap between each year’s home range to evaluate consistency of home range location between years.

Overlap of home range by mated pairs did not significantly differ from overlap of each individual with other individuals (excluding the individual’s mate; *n* = 77, *W* = 603, *P =* 0.99; Table 1). Territories overlapped a mean of 10 0 (median = 11) and 7 1 (median = 8) other radio-tagged individuals in 2011 and 2012, respectively. This demonstrates extensive overlap. However, it is not reasonable to compare differences between years because not all individuals at a site were radio-tagged. All radio-tagged birds’ ranges were also overlapped by unbanded birds. The one bird regularly radio-tracked both years retained use of 51% of its 2011 breeding range in 2012.

**Table 1. Overlap of breeding season home ranges.**

|  | **Home range overlap** | | | |
| --- | --- | --- | --- | --- |
|  | **Mated pairs** | | **All individuals (excluding mate)*** | |
|  | **2011** | **2012** | **2011** | **2012** |
| **Mean SEM** | 76 4% | 75 4% | 68 6% | 68 6% |
| **Median** | 71% | 74% | 73% | 79% |
| **Range** | 66 – 91% | 50 – 98% | 2 – 100% | 0 – 100% |
| **# of birds** | 6 (3 mated pairs) | 16 (8 mated pairs) | 25 | 30 |

*Overlap of home range by each individual with other radio-tagged individuals excluding the mate.

**References**

1. White GC and Garrott RA. Analysis of Wildlife Radio-tracking Data. San Diego, CA, USA: Academic Press; 1990.
