# Supplementary material for: Clark’s Nutcracker Breeding Season Space Use and Foraging Behavior
Source: PLoS One. 2016 Feb 16;11(2):e0149116. doi: 10.1371/journal.pone.0149116 (PMC4755556; doi:10.1371/journal.pone.0149116)
Supplement: S2 Text — (DOCX) [file pone.0149116.s009.docx]

**Variation in foraging on seed caches between years**

Clark’s nutcrackers foraged on a significantly lower proportion of cached seeds in 2011, the year after a low whitebark pine cone crop. Foraging on fewer caches may have occurred because it was more difficult to access caches below the deeper snow pack in 2011. However, Clark’s nutcrackers cache up to 59% of seeds aboveground [1], and regularly cache in exposed areas, such as steep cliffs and south facing slopes, where wind and sun prevent heavy snow accumulation [2–4]. The birds also retrieve caches from under the snow and ice [5]. I have seen a Clark’s nutcracker dig diagonally down through the snow, until its body was completely buried, then return to the surface with whitebark pine seeds (recognizable by the size; T. D. Schaming personal observation). Hutchins saw an individual peck through eight inches of ice to pull out seeds (H. E. Hutchins personal observation).

**References**

1. Lorenz TJ, Sullivan KA, Bakian AV, Aubry CA. Cache-site selection in Clark’s Nutcracker (Nucifraga columbiana). The Auk. 2011;128: 237–247.

2. Vander Wall SB, Balda RP. Coadaptations of the Clark’s nutcracker and the pinon pine for efficient seed harvest and dispersal. Ecol Monogr. 1977;47: 89–111.

3. Tomback DF. Foraging strategies of Clark’s Nutcracker. Living Bird. 1978;16: 123–160.

4. Hutchins HE, Lanner RM. The central role of Clark’s nutcracker in the dispersal and establishment of whitebark pine. Oecologia. 1982;55: 192–201.

5. Tomback DF. A late nesting attempt by Clark’s Nutcracker. Wilson Bull. 1976;88: 499–500.
